# The yeast peroxiredoxin Tsa1 protects against protein-aggregate-induced oxidative stress

**DOI:** 10.1242/jcs.144022

**Published:** 2014-03-15

**Authors:** Alan J. Weids, Chris M. Grant

**Affiliations:** The University of Manchester, Faculty of Life Sciences, Manchester M13 9PT, UK

**Keywords:** Oxidative stress, Peroxiredoxin, Protein aggregation, Mitochondria

## Abstract

Peroxiredoxins are ubiquitous thiol-specific proteins that have multiple functions in stress protection, including protection against oxidative stress. Tsa1 is the major yeast peroxiredoxin and we show that it functions as a specific antioxidant to protect the cell against the oxidative stress caused by nascent-protein misfolding and aggregation. Yeast mutants lacking *TSA1* are sensitive to misfolding caused by exposure to the proline analogue azetidine-2-carboxylic acid (AZC). AZC promotes protein aggregation, and its toxicity to a *tsa1* mutant is caused by the production of reactive oxygen species (ROS). The generation of [*rho*^0^] cells, which lack mitochondrial DNA, rescues the *tsa1* mutant AZC sensitivity, indicating that mitochondria are the source of ROS. Inhibition of nascent-protein synthesis with cycloheximide prevents AZC-induced protein aggregation and abrogates ROS generation, confirming that the formation of aggregates causes ROS production. Protein aggregation is accompanied by mitochondrial fragmentation, and we show that Tsa1 localises to the sites of protein aggregation. Protein aggregates are formed adjacent to mitochondria, and our data indicate that active mitochondria generate ROS. These data indicate a new role for peroxiredoxins in protecting against ROS that are generated as a result of protein misfolding and aggregate formation.

## INTRODUCTION

Peroxiredoxins (Prxs) are ubiquitous thiol-specific proteins, which are implicated in many cell processes and disease states. Although they are primarily characterised as antioxidants with specificity for hydroperoxides, recent data suggest that their cellular role is not simply limited to the detoxification of reactive oxygen species (ROS). Typical 2-Cys Prxs act as dimers and contain two redox-active cysteine residues that are directly involved in enzyme activity. During the peroxidation reaction, the peroxidatic cysteine residue is oxidised to a sulphenic acid, which condenses with a resolving cysteine (from the other subunit of the dimer) to form a disulphide. This disulphide is normally reduced by thioredoxin in a redox cycle that accepts electrons from NADPH. However, during periods of oxidative stress, Prxs are susceptible to the hyperoxidation of their peroxidatic cysteine residue, significantly reducing their reactivity with peroxides. This hyperoxidation is reversible by sulfiredoxins through ATP-dependent reduction (Biteau et al., 2003). Several functional gains following this paradoxical inactivation of Prxs during oxidative stress have been proposed, including hydrogen peroxide (H_2_O_2_)-based signalling and the activation of chaperone activity (Jang et al., 2004; Wood et al., 2003). More recently, the inactivation of Prxs in the fission yeast *Schizosaccharomyces pombe* was shown to redirect thioredoxin to damaged substrates and was crucial for cell viability under stress conditions (Day et al., 2012).

The budding yeast *Saccharomyces cerevisiae* contains five Prxs [thiol-specific antioxidant proteins 1 and 2 (Tsa1 and Tsa2), disrupter of telomere silencing protein 5 (Dot5), alkyl hydroperoxide reductase 1 (Ahp1) and Prx1], which differ in their target substrates and intracellular localisation (Park et al., 2000). All five are active as peroxidases, but Tsa1 plays the dominant role as an antioxidant. Cytosolic Tsa1 is the most abundant Prx in yeast and is crucial for resistance to exogenously added H_2_O_2_ (Garrido and Grant, 2002; Wong et al., 2004). Tsa1 is also required during normal aerobic growth conditions as its absence results in an increased rate of spontaneous mutations, which arise as a result of the production of endogenous ROS (Iraqui et al., 2009). Loss of *TSA1* has also been shown to increase the levels of dNTPs, resulting in increased rates of mutagenesis and genomic instability (Davidson et al., 2012; Tang et al., 2009). Tsa1 is important for longevity in yeast and is required for the lifespan extension that is normally observed in response to caloric restriction (Molin et al., 2011). The role of Prxs in protecting against genome instability appears to be evolutionarily conserved as increased malignancies are observed in mice lacking the Tsa1 homologue Prdx1 (Neumann et al., 2003).

Extensive evidence now suggests that there are many possible causal links between protein oxidation, protein misfolding and aggregate formation. Interestingly therefore, Tsa1 was proposed to act as a molecular chaperone that promotes resistance to heat shock (Jang et al., 2004). The chaperone activity involves a stress-dependent switch from low-molecular-mass species to high-molecular-mass complexes. The peroxidase function predominates in the lower-molecular-mass forms, whereas the chaperone function predominates in the high-molecular-mass complexes. The *in vivo* significance of Prx chaperone function is not clear because most Tsa1-dependent functions that have been identified to date depend on its peroxidase activity. For example, the loss of *TSA1* results in the aggregation of ribosomal proteins, indicating that Tsa1 functions to maintain the integrity of the translational apparatus (Rand and Grant, 2006). The peroxidase activity of Tsa1 is required for its ribosomal function, and Tsa1 appears to function predominantly as an antioxidant in protecting both the cytosol and actively translating ribosomes against endogenous ROS (Trotter et al., 2008). The loss of *TSA1* has also been shown to increase the frequency of *de novo* prion formation in yeast (Sideri et al., 2010).

All organisms are exposed to ROS during the course of aerobic metabolism or following exposure to radical-generating compounds. ROS cause wide-ranging damage, eventually leading to cell death. A common response to oxidative stress is a global inhibition of translation, which acts to reduce the burden of protein synthesis under potentially error-promoting conditions (Pavitt and Ron, 2012). This is important because mis-translated proteins can undergo misfolding and aggregation that can be toxic to cells. Recent evidence suggests that misfolded proteins are more susceptible to oxidation, presumably reflecting the oxidation of non-native protein structures (Dukan et al., 2000). Aminoglycoside antibiotics target ribosomes and cause mis-translation and, in *Escherichia coli*, these translational errors have been shown to result in increased protein oxidative damage. The antioxidant alkyl hydroperoxide reductase, which scavenges H_2_O_2_, reduces aminoglycoside toxicity by protecting cells from ROS-induced protein aggregation (Ling et al., 2012). We have previously shown that *tsa1* mutants are sensitive to aminoglycoside translational inhibitors, including paromomycin and hygromycin B (Trotter et al., 2008). In the current study, we show that the loss of *TSA1* also results in sensitivity to the proline analogue azetidine-2-carboxylic acid (AZC), which is used to induce the misfolding of nascent proteins. However, rather than protecting mis-translated proteins against oxidation, Tsa1 is specifically required to protect the cell against mitochondrially-derived ROS that are generated as a consequence of protein-aggregate formation.

## RESULTS

### The antioxidant activity of Tsa1 is required for resistance to AZC

**Fig. 1.**
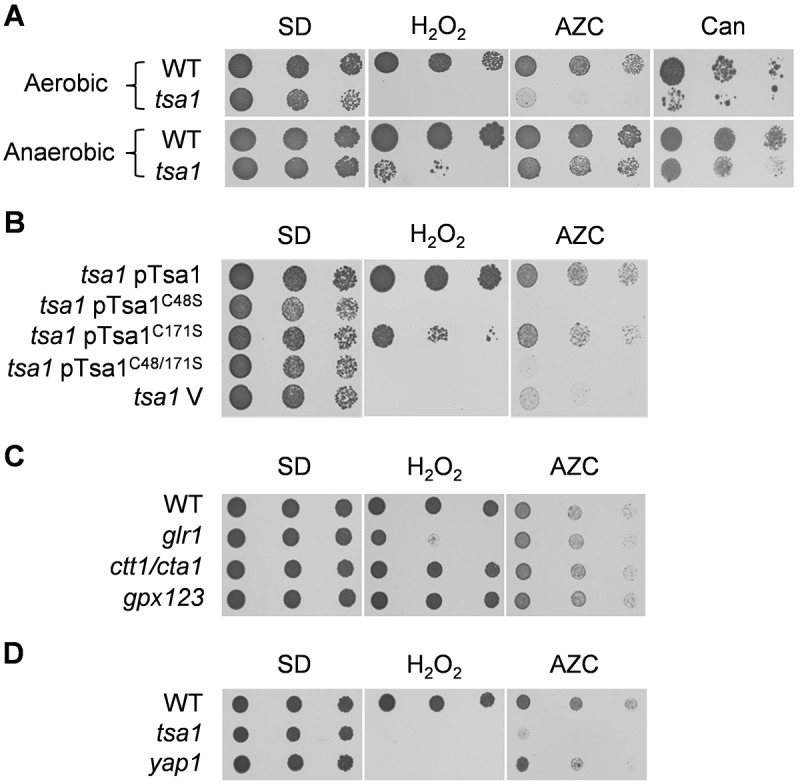
***TSA1* deletion strains are sensitive to AZC in a ROS-dependent manner.** (A) *TSA1* deletion strains exhibit aerobic but not anaerobic sensitivity to AZC. Wild-type and *tsa1*-mutant strains were grown to stationary phase and spotted onto SD medium containing H_2_O_2_ (1 mM), AZC (1 mM) or canavanine (Can) (0.2 mM). The strains were grown under either aerobic (3 days) or anaerobic conditions (six days). (B) The peroxidatic cysteine residue is essential for AZC resistance. The indicated strains were grown to stationary phase and spotted onto SD medium containing H_2_O_2_ or AZC. *tsa1 *V,* tsa1 *mutant with empty vector. (C) AZC sensitivity is not a general feature of antioxidant mutants. The indicated strains were grown to stationary phase and spotted onto SD medium containing H_2_O_2_ or AZC. (D) The AZC sensitivity of the *tsa1*-mutant strain is not due to a deficiency in Yap1 activation. The indicated strains were grown to stationary phase and spotted onto SD medium containing H_2_O_2_ or AZC.

Aminoglycosides cause mis-translation through specific binding to ribosomes, which results in increased protein misfolding. To determine whether sensitivity to growth conditions that cause protein misfolding is a common phenotype of *tsa1* mutants, we examined their sensitivity to the proline analogue AZC. AZC is competitively incorporated into proteins in place of proline, generating misfolded nascent proteins (Trotter et al., 2001). This analysis revealed that *tsa1* mutants show increased sensitivity to AZC, comparable to their sensitivity to H_2_O_2_ (Fig. 1A). Similarly, we found that *tsa1* mutants are sensitive to protein misfolding caused by the arginine analogue canavanine (Fig. 1A).

Tsa1 is thought to exhibit dual activities, functioning both as a peroxidase in the detoxification of hydroperoxides and as a molecular chaperone that protects against heat stress (Jang et al., 2004). Typical 2-Cys Prxs, such as Tsa1, are active as dimers and contain two redox-active cysteine residues that are directly involved in enzyme activity. During the peroxidation reaction, the peroxidatic cysteine residue (Cys^48^) is oxidised to a sulphenic acid, which condenses with a resolving cysteine (Cys^171^ from the other subunit of the dimer) to form a disulphide. The Cys^48^ residue of Tsa1 is therefore essential for peroxidase activity but is not required for chaperone activity (Jang et al., 2004). Accordingly, Tsa1^C48S^ and Tsa1^C48,171S^ mutants lack peroxidase activity and are sensitive to H_2_O_2_ (Fig. 1B). Although oxidised Tsa1 cannot form an intermolecular disulphide bond in the C171S mutant, the presence of the peroxidatic cysteine residue, in combination with the high abundance of Tsa1, presumably provides enough activity for partial resistance to oxidants (Fig. 1B). Tsa1 cysteine mutants displayed the same pattern of sensitivity to AZC as to H_2_O_2_, indicating that it is the peroxidase activity of Tsa1 that is important for AZC tolerance.

Sensitivity to AZC does not appear to be a general phenotype of mutants that are deficient in antioxidants because yeast strains lacking catalases (*CTT1*, *CTA1*), glutathione reductase (*GLR1*) and glutathione peroxidases (*GPX1–GPX3*) showed no increase in sensitivity to AZC (Fig. 1C). Notably, the *glr1* mutant showed a similar sensitivity to H_2_O_2_ compared with the *tsa1* mutant but is not sensitive to AZC. Additionally, we examined the AZC sensitivity of a mutant lacking the Yap1 transcription factor, which is a major regulator of the oxidative stress response in yeast (D'Autréaux and Toledano, 2007). Tsa1 is known to interact with Yap1 and to be able to act as an H_2_O_2_ receptor and relay the signal to Yap1 in the W303 yeast strain used in our studies (Tachibana et al., 2009). However, the *yap1* mutant was only partially sensitive to AZC and was not as sensitive to AZC as the *tsa1* mutant, which would be expected if the AZC sensitivity of the *tsa1* mutant depended on the interaction of Tsa1 with Yap1 (Fig. 1D). Furthermore, a *tsa1* mutant in another yeast-strain background (the BY strain), which does not require Tsa1 for Yap1 activation, is also sensitive to AZC (data not shown). Taken together, these data suggest that the antioxidant activity of Tsa1 is specifically required to protect against the toxicity of AZC.

### Mitochondrially generated ROS account for the AZC-sensitivity of *tsa1* mutants

**Fig. 2.**
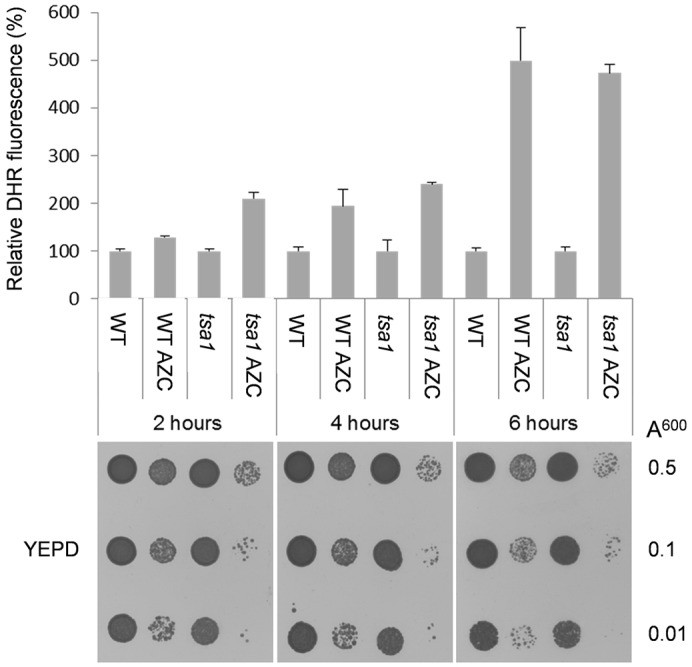
**AZC treatment causes ROS formation.** The strains were grown to exponential phase in SD medium before treatment with AZC (5 mM). Following incubation with DHR123, cell extracts were prepared and their fluorescence was measured (mean±s.d., *n* = 3). To assess the cell viability at each of the indicated timepoints, samples from each condition were adjusted to equal OD_600_ values and were serially diluted and spotted onto YEPD media.

Given that the peroxidase activity of Tsa1 appears to be important for the tolerance to AZC, we tested whether ROS are generated following exposure to AZC, which might account for the sensitivity of *tsa1* mutants to AZC. For this analysis, we used dihydrorhodamine 123 (DHR123), which has been used extensively for the *in vivo* detection of ROS (Honda et al., 2012; Swalwell et al., 2012). Upon oxidation, DHR123 is converted into rhodamine, which emits a detectable fluorescent signal. Over a 6-h timecourse, we observed increasing ROS generation in both the wild-type and *tsa1*-mutant strains exposed to AZC compared with untreated control cultures (Fig. 2). ROS levels were significantly higher in the *tsa1* mutant compared with the wild type following 2 h of AZC exposure. This difference was lost at later timepoints, with ROS levels increasing approximately four- to fivefold following 6 h of AZC treatment in both the wild-type and *tsa1*-mutant strains. We examined cell viability over this timecourse by spotting cultures onto yeast extract peptone dextrose (YEPD) plates (Fig. 2). The *tsa1* mutant was very sensitive to AZC and we observed greatly reduced viability within 2 h in the *tsa1* mutants compared with the wild-type strain. Hence, ROS might not accumulate at higher levels in the *tsa1* mutant at later timepoints owing to the loss of cell viability. If ROS generation accounts for the sensitivity of *tsa1* mutants to AZC, we reasoned that anaerobic growth conditions should rescue the growth defect. In agreement with this idea, anaerobic growth conditions strongly rescued the AZC sensitivity of the *tsa1* mutant (Fig. 1A). The sensitivity of the *tsa1* mutant to canavanine could also be rescued under anaerobic conditions (Fig. 1A).

**Fig. 3.**
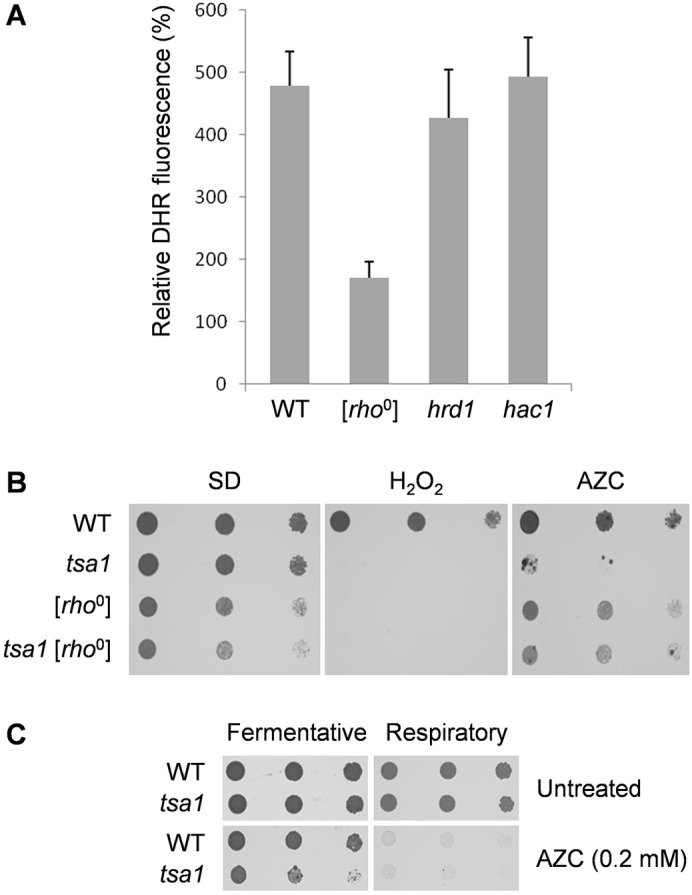
**AZC treatment induces mitochondrial ROS formation.** (A) The generation of ROS is abrogated in [*rho*^0^] strains. The indicated strains were grown to mid-exponential phase in SD medium, before treatment with AZC (5 mM) for 6 h. Following incubation with DHR123, cell extracts were prepared and their fluorescence was measured (mean±s.d., *n* = 3). (B) Transformation of the *tsa1* mutant strain to [*rho*^0^] rescues its sensitivity to AZC but not to H_2_O_2_. The indicated strains were grown to stationary phase and spotted onto SD medium containing AZC (2 mM) or H_2_O_2_ (1 mM). (C) AZC toxicity is exacerbated under respiratory conditions. The indicated strains were grown to stationary phase and spotted onto SD medium (fermentative) or SGE medium (respiratory), with or without AZC (0.2 mM).

We addressed the potential sources of AZC-induced ROS using a candidate approach. Oxidative phosphorylation, which occurs during mitochondrial respiration, is thought to be the main source of ROS in eukaryotic cells (Murphy, 2009). Additionally, the use of oxygen as a terminal electron acceptor during oxidative protein folding means that the endoplasmic reticulum (ER) is also a potential source of ROS (Tu and Weissman, 2004). In particular, sustained ER stress due to prolonged activity of the unfolded protein response (UPR) has been shown to cause an accumulation of ROS, whereas ER-associated protein degradation (ERAD) acts to prevent ROS generation by preventing misfolded proteins from accumulating in the ER (Haynes et al., 2004). We therefore measured ROS generation in mutants lacking mitochondrial function [*rho*^0^] and in mutants defective in ERAD (*hrd1*) or the UPR (*hac1*) (Fig. 3A). Strikingly, ROS generation was unaffected in *hrd1* or *hac1*mutants but was strongly abrogated in [*rho*^0^] mutants. This suggests that mitochondria are the major source of ROS following AZC stress. Given that the mitochondria appear to account for the majority of the ROS that are generated in response to AZC exposure, we examined whether disrupting mitochondrial function could reduce the AZC sensitivity of a *tsa1* mutant (Fig. 3B). The absence of electron transport ([*rho*^0^] state) was found to improve the AZC tolerance of the *tsa1* mutant but did not affect the sensitivity of the mutant to oxidative stress caused by H_2_O_2_. Furthermore, growth on non-fermentable carbon sources (where mitochondrial respiration is required) was strongly inhibited in the presence of low concentrations of AZC (Fig. 3C). These data indicate that the sensitivity of *tsa1* mutants to AZC is due to the requirement for Tsa1 antioxidant function to protect against mitochondrially generated oxidative stress.

### AZC induces protein aggregation

**Fig. 4.**
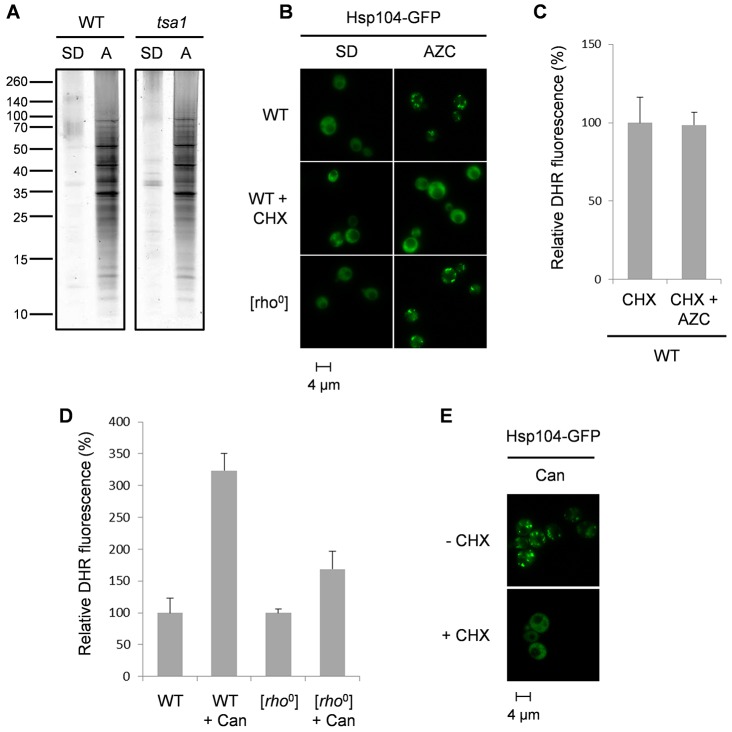
**AZC stress causes protein aggregation.** (A) AZC treatment results in similar patterns of protein aggregates in wild-type and *tsa1*-mutant strains. Protein aggregates were isolated from wild-type and *tsa1*-mutant strains following 2 h of AZC treatment (5 mM) and were analysed by SDS-PAGE and silver staining. A, AZC treatment. (B) Hsp104–GFP forms multiple foci following AZC stress, and conversion to [*rho*^0^] does not affect the foci formation. The formation of Hsp104 foci following AZC treatment requires active protein synthesis because it is prevented by growth with cycloheximide (10 µg/ml, CHX). (C) The generation of ROS following AZC treatment requires active protein synthesis. Strains were grown to exponential phase in SD medium before treatment with CHX (200 µg/ml) or CHX plus AZC (5 mM) for 6 h. (D) Canavanine treatment results in ROS generation, which is abrogated in [*rho*^0^] cells. The indicated strains were grown to mid-exponential phase in SD medium, before treatment with canavanine (5 mM) for 6 h. (E) The formation of Hsp104 foci following canavanine treatment requires active protein synthesis.

Given the well-established links between mis-translation and protein misfolding, we examined whether AZC promotes protein aggregation, which might account for the sensitivity of *tsa1* mutants to AZC. Insoluble protein aggregates were prepared as described previously and were analysed using SDS-PAGE (Rand and Grant, 2006; Trotter et al., 2008). AZC was found to significantly increase protein aggregation in both the wild-type and *tsa1*-mutant strains (Fig. 4A). A similar pattern of aggregated protein was observed in the wild type and *tsa1* mutant, suggesting that increased levels of aggregation following AZC exposure do not explain the sensitivity of *tsa1* mutants to AZC. The sites of protein aggregation can be detected using fluorescently tagged chaperones such as heat shock protein 104 (Hsp104, a protein disaggregase) (Erjavec et al., 2007; Lee et al., 2010). Similarly, Hsp104–GFP was found to form foci in response to AZC-induced stress (Fig. 4B). Protein aggregation was still induced by AZC in a [*rho*^0^] mutant, confirming that this mutant did not generate a reduced amount of ROS simply by preventing protein aggregation (Fig. 4B).

To confirm directly that it is the misincorporation of AZC into proteins and the resulting protein aggregation that cause mitochondrial ROS production, rather than any other potential effect of AZC stress, we treated cells with cycloheximide, which is a potent inhibitor of protein synthesis. We confirmed that cycloheximide prevented AZC-induced aggregate formation by showing that Hsp104–GFP did not form foci in response to AZC in the presence of cycloheximide (Fig. 4B). Cycloheximide treatment also prevents AZC-induced ROS formation, further confirming that it is the misincorporation of AZC into proteins and the resulting protein aggregation that is the cause of ROS production (Fig. 4C). To confirm that these effects are not specific to AZC, we examined protein aggregation in response to canavanine stress. ROS assays confirmed that canavanine promotes ROS generation that requires mitochondrial function, as ROS generation is abrogated in a [*rho*^0^] mutant (Fig. 4D). Hsp104–GFP formed foci in response to canavanine stress and, again, this was dependent on nascent protein synthesis as foci formation was prevented by cycloheximide treatment (Fig. 4E).

### Tsa1 colocalises with sites of protein aggregation adjacent to active mitochondria

**Fig. 5.**
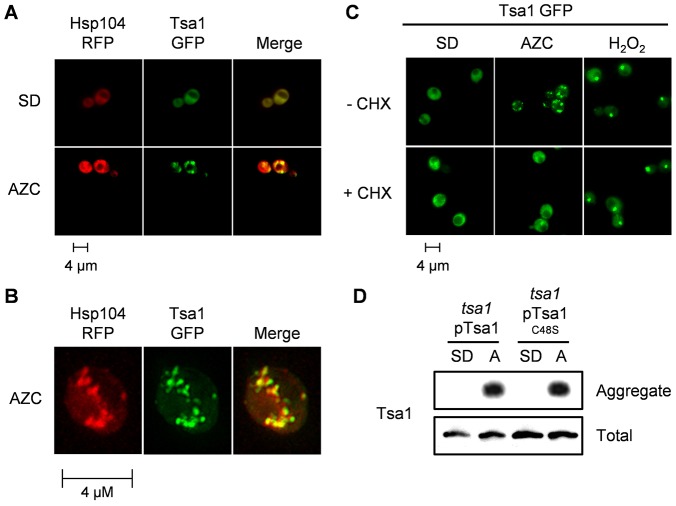
**Tsa1 colocalises with sites of protein aggregation.** (A) Tsa1 colocalises with Hsp104 following AZC stress. A wild-type strain coexpressing Tsa1–GFP and Hsp104–RFP was grown to mid-exponential phase before AZC (5 mM) treatment. (B) Confirmation of the colocalisation of Tsa1 and Hsp104. Images were acquired on a Deltavision restoration microscope, deconvolved and presented as a maximum-intensity projection from optical sections. (C) The formation of Tsa1–GFP foci in response to AZC, but not H_2_O_2_, requires nascent-protein synthesis because it is abrogated by cycloheximide (CHX) treatment. (D) Cys^48^ is not required for the localisation of Tsa1 to insoluble protein aggregates. Insoluble protein aggregates were prepared as in Fig. 4A and Tsa1 was detected by western blot. A, AZC treatment.

To examine the cellular distribution of Tsa1, we monitored the localisation of a GFP-tagged version of Tsa1. Tsa1–GFP displayed diffuse cytoplasmic fluorescence during normal growth conditions, whereas 30 min of AZC treatment caused the redistribution of Tsa1–GFP to multiple distinct foci (Fig. 5A). This response is not shared with another highly abundant peroxiredoxin, Ahp1, which displayed diffuse fluorescence both under normal conditions and during AZC stress (data not shown). We confirmed that Tsa1 localises to the sites of protein aggregation by showing that, in response to AZC stress, Hsp104–RFP formed foci that exhibited significant overlap with Tsa1–GFP (Fig. 5A). This overlap was confirmed as colocalisation through the acquisition and projection of a *z*-stack of optical sections (Fig. 5B). The formation of Tsa1 foci was dependent on nascent-protein synthesis because cycloheximide prevented the formation AZC-induced foci (Fig. 5C). To rule out the possibility that AZC causes protein aggregation and ROS generation in a manner independent of its effect on protein synthesis, we examined protein aggregation in response to oxidative stress that was generated by exposure to H_2_O_2_. H_2_O_2_ caused Tsa1 to accumulate in foci but this did not depend on protein synthesis because it was unaffected by cycloheximide treatment (Fig. 5C). Hence, AZC does not cause protein aggregation due to ROS generation independent of its effect on protein misfolding and aggregation. Given that the Cys^48^ residue of Tsa1 is required for resistance to AZC, we examined whether the Tsa1^C48S^ mutant was able to localise to the aggregate fractions following AZC stress. We found that both the wild-type Tsa1 and mutant Tsa1^C48S^ were detectable at similar levels in insoluble protein aggregates following AZC treatment but both were undetectable in aggregate fractions isolated under normal conditions (Fig. 5D). This supports the idea that it is the Tsa1 peroxidase activity that is required at sites of protein aggregates, as the peroxidatic Cys^48^ residue of Tsa1 would be free to detoxify peroxides rather than being required for the localisation of Tsa1 to the sites of aggregation.

**Fig. 6.**
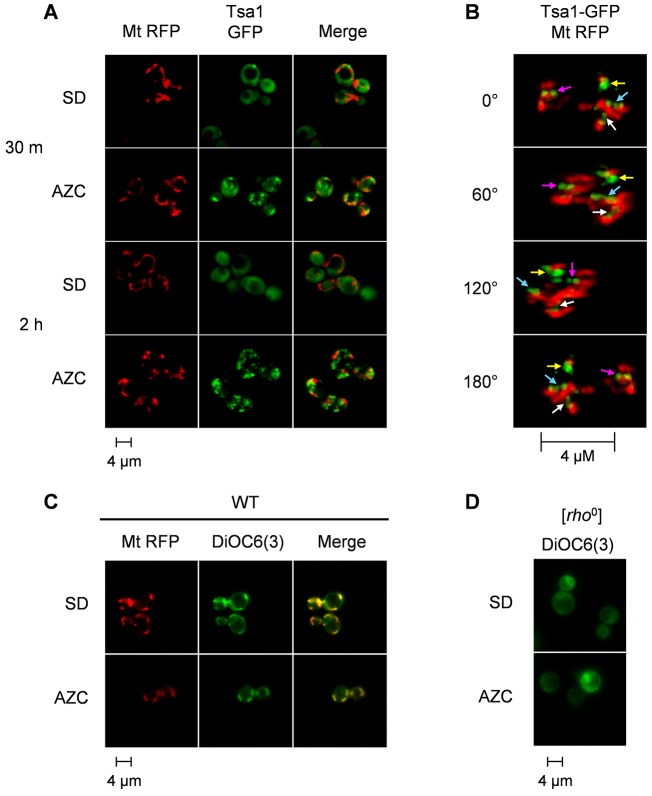
**Sites of protein aggregation are adjacent to active mitochondria.** (A) Mitochondria undergo fragmentation following AZC treatment. A wild-type strain coexpressing Tsa1–GFP and mt–RFP was grown to mid-exponential phase before AZC (5 mM) treatment. Images were collected at 30 min and 2 h. (B) The sites of protein aggregation localise adjacent to mitochondria. The cells were fixed with formaldehyde and visualised on a Deltavision restoration microscope. A 3D projection was generated and snapshots of four angles up to 180° around the *y*-axis (0°, 60°, 120° and 180°) were processed. Four major Tsa1 granules are traceable through the angles, as indicated by the coloured arrows. (C) Mitochondrial membrane potential is maintained during AZC stress. Following AZC treatment, cells were incubated with DiOC_6_(3). (D) No DiOC_6_(3) staining was detected in a [*rho*^0^] mutant.

Given that the mitochondria are the main source of AZC-induced ROS, we examined mitochondrial morphology using a mitochondrially targeted RFP probe (mt-RFP). During normal non-stressed conditions, the mitochondria appeared as a network of tubules (Fig. 6A). After 30 min of AZC treatment, the mitochondrial morphology was consistent with that of a wild-type strain. However, following 2 h of AZC treatment, the mitochondria appeared as multiple fragmented structures that were distributed throughout the cells (Fig. 6A). The formation of Tsa1 puncta was visible within 30 min and preceded mitochondrial fragmentation. Interestingly, Tsa1 puncta appeared to form adjacent to fused mitochondria and remained next to the mitochondria even after mitochondrial fragmentation (Fig. 6A). This was most apparent when visualised as a 3D projection using a Deltavision deconvolution microscope (Fig. 6B). Mitochondrial function was assessed by using the membrane-potential-sensitive stain 3,3′-dihexyloxacarbocyanine iodide [DiOC_6_(3)], which stains mitochondrial membranes that have a membrane potential. DiOC_6_(3) was found to show a similar pattern of mitochondrial staining to the mitochondrially targeted RFP probe (Fig. 6C). By contrast, no mitochondrial membrane staining was detected in a [*rho*^0^] mutant (Fig. 6D). These data indicate that, even though mitochondria are fragmented following AZC stress, they maintain a membrane potential that is indicative of active mitochondria.

## DISCUSSION

Both newly synthesised and stress-denatured proteins require a complex network of chaperones to reach their final native states efficiently. In the absence of chaperones, many proteins form non-native protein structures and partially folded intermediates that tend to aggregate. These protein aggregates can cause toxicity by irreversibly removing proteins from their productive folding pathways, and they are associated with various neurodegenerative diseases (Kim et al., 2013). Toxicity can also be caused by the aggregates interacting with and damaging other cell components, and by the depletion of cellular chaperones. ROS have also been implicated in protein aggregation and the pathogenesis of several neurological disorders. This includes studies in model organisms, which have shown that partially misfolded and mis-translated proteins are more susceptible than correctly folded proteins to oxidation and protein aggregation (Dukan et al., 2000; Ling et al., 2012). Oxidative damage has been shown to influence the formation of toxic oligomers that underpin several major neurodegenerative diseases. In Alzheimer's disease, ROS affect the regulation of the processing enzymes of amyloid precursor protein (APP), which convert APP to the toxic Aβ_1–42_ oligomers (Cai et al., 2011). The toxic aggregated form of α-synuclein, which is associated with Parkinson's disease (PD), has been shown to oligomerise upon methionine oxidation (Leong et al., 2009). Furthermore, cysteine oxidation of the mutant huntingtin protein that causes Huntington's disease (HD) promotes the formation of stable oligomers (Fox et al., 2011). The Tsa1 peroxiredoxin has previously been implicated in protecting cells against ROS-dependent protein aggregation because it suppresses the oxidative-stress-induced *de novo* formation of prions in yeast (Sideri et al., 2010). Our current study indicates that, rather than oxidative stress promoting protein-aggregate formation, the protein aggregates themselves can promote ROS generation following the misfolding of nascent protein.

The removal of molecular oxygen or the disruption of mitochondrial function rescued the sensitivity of the *tsa1* mutant to AZC, indicating that the toxicity is due to ROS formation, rather than due to any effect on protein homeostasis through protein-aggregate formation. Similar reductions in the activity of the mitochondrial electron transport chain in higher eukaryotes have been implicated in the extension of lifespan. However, this does not necessarily depend on reduced ROS formation and oxidative stress because increased longevity due to reduced mitochondrial activity requires the mitochondrial UPR (Durieux et al., 2011). This protein homeostasis mechanism has not yet been described in yeast, but it is possible that similar mitochondrial-stress systems are involved in AZC toxicity. In this study, ROS formation was measured, confirming that the AZC-induced increases in ROS are detected in both the wild-type and *tsa1*-mutant strains. AZC stress is not toxic to the wild-type strain under the conditions used in our studies; hence it is the loss of the antioxidant activity of Tsa1 that results in the sensitivity to AZC-induced ROS. AZC is a proline analogue that is competitively incorporated into nascent proteins (Trotter et al., 2001). AZC incorporation alters the conformation of the polypeptide backbone, which results in decreased thermal stability and misfolding. Our current data indicate that AZC incorporation promotes significant levels of protein aggregation. Hsp104 is a stress-inducible protein disaggregase that has been used as an *in situ* marker for protein aggregation (Erjavec et al., 2007; Glover and Lindquist, 1998). We detected colocalisation of Tsa1 and Hsp104, indicating that Tsa1 moves to the sites of protein aggregation in response to AZC stress. Increasing the cellular misfolding load with AZC resulted in the accumulation of aggregates, and our data indicate that the generation of ROS is a by-product of these protein deposits. Tsa1 appears to act as a specific antioxidant that can detoxify the ROS that are associated with protein aggregation because mutants lacking other major antioxidants did not show an increase in AZC sensitivity. One possibility is that Tsa1 localises to protein aggregates in order to detoxify the ROS that are locally generated at these insoluble protein deposits. This view is supported by the finding that the peroxidatic cysteine residue was not required for the localisation of Tsa1 to protein aggregates, thus keeping the catalytic residue free to detoxify peroxides.

Many neurodegenerative diseases are accompanied by the appearance of protein aggregates that are characteristic for each particular disease. An increasing body of evidence has implicated ROS generation in the toxicity of these diseases, although the sources of the ROS are often not well defined. There is some evidence that the protein aggregates themselves can act as sources of free radicals, dependent on metal-catalysed reactions (Allsop et al., 2008). Additionally, the disruption of antioxidant systems, such as the glutathione system, has been proposed to cause oxidative stress and cell death in HD (Li et al., 2010). Our data indicate that the mitochondria are the main source of ROS following AZC stress because the loss of mitochondrial function largely prevented ROS generation. Interestingly, the sites of AZC-induced protein aggregation predominantly occurred adjacent to mitochondria. Mitochondrial dysfunction is thought to be a key event in some neurodegenerative diseases, and mutant huntingtin has been shown to trigger mitochondrial fragmentation by stimulating DRP1, the mitochondrial fission GTPase dynamin-related protein-1 (Song et al., 2011). Similarly, α-synuclein causes mitochondrial fragmentation that is implicated in the pathogenesis of PD (Nakamura et al., 2011).

Mitochondrial fission and fusion are normal processes in mitochondrial dynamics. However, alterations in these processes and the accompanying mitochondrial fragmentation have been implicated in cell death during various stress conditions (Scheckhuber et al., 2007; Youle and Karbowski, 2005). Mitochondrial fragmentation acts to separate defective organelles from the mitochondrial network, with subsequent degradation by mitophagy. In this study, AZC stress and ROS generation were accompanied by mitochondrial fragmentation. It is not clear why increased mitochondrial fragmentation is observed in response to AZC stress. The wild-type yeast cells were able to survive the AZC stress used in our study, and it is possible that more elevated AZC stress, or AZC stress in chaperone mutants that cannot detoxify protein aggregates, would cause the affected cells to undergo mitochondrial fragmentation as a precursor to apoptosis. Despite the mitochondrial fragmentation that occurred in response to AZC stress, the mitochondria remained metabolically active. This suggests that the AZC-induced fragmentation of mitochondria reflects a change in the energy requirements of the cell. This is in line with accumulating evidence that fluidity in mitochondrial morphology (fusion and fission) is required to maintain adaptive bioenergetics (Liesa and Shirihai, 2013). For example, a loss of DRP1 inhibits mitochondrial fragmentation and results in defects in mitochondrial bioenergetics, including a decreased rate of ATP synthesis and a reduction in the level of ATP (Benard et al., 2007; Parone et al., 2008). Furthermore, mitochondrial fragmentation has been shown to result in ROS overproduction and increased respiration (Yu et al., 2006). In our scenario, given the energy-requiring processes that are needed to detoxify and repair misfolded and damaged proteins, it is possible that the elevated generation of ROS reflects the need for increased mitochondrial function. Such an increase in function might be required to provide the necessary energy to remove protein aggregates, as well as to replace the proteins that are sequestered within the aggregates. Although protein aggregates serve to sequester misfolded and aberrant proteins, their toxicity has long been implicated in many disease states. Our current data suggest that mitochondrial ROS generation might account for one key aspect of this toxicity, which is normally countered by the antioxidant activity of peroxiredoxins.

## MATERIALS AND METHODS

### Yeast strains and plasmids

The wild-type strain W303 (*MATa ura3-52 leu2-3 leu2-112 trp1-1 ade2-1 his3-11 can1-100*) and its isogenic derivatives deleted for *TSA1* (*tsa1::LEU2*, *tsa1*::URA3), *GLR1* (*glr1::TRP1*), *GPX1,2,3* (*gpx1::TRP1 gpx2::URA3 gpx3::HIS3*), *YAP1* (*yap1*::*HIS3*) and catalases (*ctt1::URA3 cta1::URA3*) have all been described previously (Collinson et al., 2002; Grant et al., 1996; Grant et al., 1998; Rand and Grant, 2006). Strains deleted for *HRD1* (*hrd1::KanMX4*) and *HAC1* (*hac1::KanMX4*) were constructed using standard yeast techniques. Respiratory incompetent [*rho*^0^] versions of the wild-type and *tsa1* mutant strains were created using treatment with ethidium bromide. GFP-tagged (*TSA1–GFP*, *AHP1–GFP*) strains were obtained from the yeast GFP clone collection supplied by Life Technologies. RFP-tagged Hsp104 (pRP1186–Hsp104–mRFP) is described elsewhere (Lee et al., 2010) and mtRFP, which was used to RFP-label mitochondria, was a kind gift from Campbell Gourlay (University of Kent, UK).

### Growth conditions

Strains were grown in minimal selective dropout (SD) medium [0.17% (w/v) yeast nitrogen base without amino acids, 5% (w/v) ammonium sulphate and 2% (w/v) glucose] supplemented with the appropriate amino acids and bases at 30°C with agitation at 180 rpm. For growth on non-fermentable carbon sources, strains were grown on selective glycerol ethanol (SGE) containing 3% (v/v) glycerol and 1% (v/v) ethanol. Media were solidified by the addition of 2% (w/v) agar. Stress sensitivity was determined by growing the cells to stationary phase and spotting diluted cultures (A_600_ = 1.0, 0.1 and 0.01) onto agar plates containing various concentrations of inhibitor. Growth was monitored after 3 days. For anaerobic growth conditions, SD medium was supplemented with 0.1% (v/v) Tween-80 and 30 mg.l^−1^ ergosterol, and the plates were maintained in an anaerobic jar containing a gas-generating kit (GasPak EZ; Becton, Dickinson & Co). Growth was monitored after 6 days.

### Analysis of insoluble protein aggregates

Insoluble protein aggregates were isolated as described previously (Rand and Grant, 2006), with the minor adjustments that are described below. Cell breakage was achieved by sonication (Sonifier 150, Branson; 8×5 s, Level 4) and the samples were adjusted to equal protein concentrations before the isolation of protein aggregates. The insoluble fractions were resuspended in detergent washes by sonication (4×5 s, Level 4). The insoluble fractions were resuspended in reduced protein loading buffer, separated by reducing SDS/PAGE (12% gels) and visualised by silver staining with the Bio-Rad silver stain plus kit. For western blot analysis, protein extracts were electrophoresed under reducing conditions and electroblotted onto polyvinylidene difluoride (PVDF) membrane (Amersham Pharmacia Biotech). Bound antibody was visualised by chemiluminescence (ECL, Amersham Pharmacia Biotech).

### Measurement of intracellular ROS

Intracellular ROS were measured at intervals of 2, 4 and 6 h following AZC (5 mM) treatment as described previously (Rand and Grant, 2006), with the minor amendments described below. Dihydrorhodamine 123 (DHR123, Sigma-Aldrich) was used to assess the presence of ROS and the 485/528 nm values were adjusted to A_280_ protein absorbances to generate the relative DHR123 fluorescence values.

### Fluorescence microscopy

The cells were grown to mid-exponential phase in SD medium before treatment. The cells were washed and immobilised on 10% poly-L-lysine-coated slides and visualised on an Olympus BX51 upright microscope using a ×100/NA1.30 UPlanFLN objective, and images were captured using a Coolsnap ES camera (Photometrics) with MetaVue Software (Molecular Devices). The images were taken in one representative focal plane and were processed and analysed using ImageJ (http://rsb.info.nih.gov/ij). For colocalisation and 3D projections, the images were acquired on a Delta Vision (Applied Precision) restoration microscope using a ×100/NA1.40 Plan Apo objective and fluorescein isothiocyanate (FITC) and Texas Red band pass filters from the Sedat filter set (Chroma). The images were collected using a Coolsnap HQ (Photometrics) camera with a *Z* optical spacing of 0.2 µm. The raw images were then deconvolved using the Softworx software, and the maximum intensity or 3D projections (for the latter, 180° around the *y*-axis) of these deconvolved images are shown in the results. For DiOC_6_(3) staining of mitochondria, cells were grown to mid-exponential phase before treatment. The cells were incubated in 2.5 ng/ml DiOC_6_(3) (Life Technologies) for 5 min before washing and visualisation.
